# The Relation of Thorough Mouth Treatment of Food to the Affections of the Teeth1Read before the Harvard Odontological Society, April 20, 1905.

**Published:** 1905-06

**Authors:** Horace L. Howe

**Affiliations:** Boston, Mass.


### THE RELATION OF THOROUGH MOUTH TREATMENT OF FOOD TO THE AFFECTIONS OF THE TEETH.[1]

BY DR. HORACE L. HOWE, BOSTON, MASS.

THAT the teeth and adjoining tissue are influenced by the general health is a fact long known to science.

Every truth has its corollary.

Thus the general health of the individual is, to a certain extent, influenced by the teeth; in that they are essential for the proper mouth treatment of food, which goes to build up tissue upon which every faculty of mind and body is dependent.

We cannot ignore the function of any part of the body, or any faculty of the mind, without impairing, to a degree, the total capacity of body or mind.

Every part is dependent, to some extent, upon other parts, and has its relation to the whole.

If we attend to the voluntary part of nutrition, or the stage of digestion before the food goes to the stomach, nature will perform the involuntary part to perfection.

---

[1] Read before the Harvard Odontological Society, April 20, 1905.

It is my object to-night not to give an extended account of the affections of the teeth, but simply to agitate the subject of nutrition as related to the teeth.

To do this best I will refer to a book written by Mr. Horace Fletcher, and called "The A, B, and Z of Our Own Nutrition."

Mr. Fletcher is startling the medical world with his discoveries regarding right living, and is devoting his life and means in telling the world of his deductions.

That he has searched out the cause of diseases arising from perverted nutrition is acknowledged and accepted by the most eminent physiologists in this country and in Europe.

To show the appreciation of Mr. Fletcher's discoveries by the medical profession, I will quote the following from a letter written him by Dr. J. H. Kellogg, of the Battle Creek Sanitarium, who says, "It is a remarkable spectacle that these great men, these learned professors and scientists and army medical men, should be co-operating so enthusiastically with a layman to learn the true philosophy of life, but it has always been so. The great discoveries have not been made by great scientists and great doctors, but by men whose minds were above the bias of prescribed education, and who were able to learn from the great book of nature."

Mr. Fletcher's discovery is as simple as many great discoveries are, and is this: That we should chew our food until it becomes practically liquefied and of alkaline reaction, by means of our saliva.

In criticism, some say that Mr. Fletcher places too much importance upon the function of mastication, and that some people are troubled with hypochlorhydria or too little hydrochloric acid in the stomach.

For a moment let us consider the process of nutrition. All our food is acid in a varying degree. Our saliva neutral,—but alkaline when we masticate,—our gastric juice acid, and so on, alternating with the other digestive fluids. We understand by Anstie's well-known law—*i.e.*, "glands exciting alkaline fluids are stimulated by the presence of acids"—and the reverse—"glands exciting acid fluids are stimulated by the presence of alkalines"—that the natural stimulus for the gastric glands would be alkalines; that is, the food, after it has been made alkaline by the saliva. If we swallow our food without sufficient chewing, the gastric glands are thus deprived of their natural stimulus, which perhaps may account for the state of hypochlorhydria.

Is it surprising that our stomachs become sour, as the expression is, and rebel, and assimilation and metabolism is affected, when we swallow our food (artificially acid, possibly) without proper mastication? The expression of this rebellion may be slight and soon overcome, but if the abuse goes on disease is sure to follow.

In my own case severe headaches were the forms in which nature showed the result of my neglect. These have been completely eradicated by strict attention to the mouth-treatment of my food.

Let us trace the process of digestion from infancy. Naturally the child is nourished by the human milk, which is normally alkaline in reaction. This is taken directly into the stomach. Thus the gastric glands have their natural stimulus,—*i.e.*, alkaline milk. As the temporary teeth erupt, the salivary glands develop. As the child is given food, the least acid foods first (cow's milk, eggs, crackers, etc.), the salivary glands become equal to the task of converting them into an alkaline solution. If the task be too great, then the trouble begins.

We have all noticed that those who are troubled by pyorrhœa alveolaris have the so-called "general uric acid diathesis," and are also troubled with pains in the joints of the extremities, or gout, rheumatism, constipation, etc.

Dr. Michaels, of Paris, in his very scientific treatise upon saliva, says, "The reaction of the blood to the ordinary agents (litmus, for example) is normally alkaline, but if we study the distribution of acids and bases of the *blood plasma*, we see that the reaction is really acid (Gautrelet, Drouin, Hugorenq), and this acidity is increased and the blood may become decidedly acid by the accumulations of acid waste products, which are not eliminated; the *secretions* and *excretions* becoming then of acid reaction."

This would lead us to believe that the serumal deposits upon the teeth, in cases of alveolar pyorrhœa are salts or urates from the blood, produced by the overacid condition of the system.

The attachments of the teeth to the alveoli are now recognized as true joints. Why is it not reasonable to suppose that deposits might be formed in these joints from the same cause as in the joints of the extremities?

Furthermore, it has been my observation that children who are troubled with sour stomachs and bilious attacks are apt to have present the rapid, soft, white caries so destructive to teeth.

*Are not these diseases of the teeth, both caries and pyorrhœa alveolaris, brought on by the absence of the normal amount of alkaline saliva, locally in the mouth and systemically by the absence of nature's neutralizing fluid in the stomach?* Tests that I have made seem to bear out this theory.

Since becoming especially interested in this subject I have tested my own saliva and that of many of my patients for acidity. There are many factors that vary the reaction, as we can see by Dr. Michaels's observation. It seems to be normally neutral, but becomes alkaline with the muscular movements of chewing, whether food be present or whether we chew but rubber. It is less alkaline in the morning after sleeping; the muscles of the tongue and jaws being then at rest. It has been observed that it becomes acid after extreme bodily fatigue. In my own case, when I do not feel well my saliva is decidedly acid.

We all know of Gladstone's health, and of his invariable rule of chewing thirty-two times before he swallowed his food.

The remedy for these difficulties that I have pointed out seems to be had in chewing thoroughly the food that we take, thus using Nature's method of neutralizing the system.

However, "reason has but little show against intrenched habit," hence it requires some strength of mind to overcome habit in order to give our food thorough mouth treatment.

It is true that people live, perhaps in good health, without paying particular attention to the voluntary part of digestion; if such be the case, however, the balance is maintained by getting rid of the excess of acids, by perspiration, or by living upon the least acid foods, as eggs, milk, etc. For instance, the Japanese, who live chiefly upon a diet of fish, rice, and other vegetables, are particularly healthy and energetic.

Those who have become vegetarians in this country notice a great improvement in general health and also in the health of the teeth and gums.

The influence of natural living upon the development and condition of the teeth is nicely shown by Dr. Campbell, of London, England. He divides the evolutionary career of man into five stages.

1. The anthropoid stage; with raw vegetable and animal food. Carious teeth were then unknown.

2. The pre-cooking stage, or the stage before the human race used fire for cooking. No caries of the teeth present.

3. The pre-agricultural period, to which period the aboriginal Australians and Bushman still belong and are available for study, with dental caries rare, and chiefly met in the third molars.

4. The early agricultural age, with dental caries more frequent.

5. The late agricultural period, or the time in which we of to-day live.

The chief characteristic of the food of to-day is its extreme softness, due to agriculture and cooking; therefore it requires little mastication. Because of the physiological law, "that the use of a part tends to stimulate its growth and development, and disuse to lack of development and atrophy," the teeth and jaws are less developed and more diseased to-day than in the previous ages.

In this same way Dr. Campbell very aptly places the cause of *adenoids and resulting evils, among them irregular teeth, to insufficient stimulation of the blood in the jaws from lack of exercise of the muscles of mastication.*

In studying the skulls of the early Indians, Hawaiians, and Peruvians at the Peabody Museum, I have noticed the comparative absence of caries and irregularity about the teeth. The greater number have good, sound teeth, much worn, but no *distal occlusion* which accompanies adenoids and undeveloped jaws.

The Ancients used to blame their stomachs for all their ills. Possibly the blame was justly placed, and the word hypochondriac (stomach region) and its modifications may rightly express the truth.

The real influence that lack of thorough mouth-treatment of food and perverted nutrition has upon all diseases, both mental and physical, is fast being made clear by the great scientists of the medical profession. For instance, Dr. Haig, the well-known expert on diet, points out that laziness, stupidity, alcoholism, and other effects that are much against the poor are largely due to excessive meat eating, and estimates that in seventy-five per cent. of the cases of insanity, cancer, debility, and degeneration generally, improper food and habits of eating are to blame for the patients' condition."

Medical history reveals the fact that the greatest physicians have used medicines only to soothe or alleviate suffering. They tried to impress their patients with the importance of proper feeding, exercising, fresh air, etc., whereas the small ones of the profession have used medicine with the idea of curing, and have neglected and overlooked the natural means. As every faculty of the mind and body grows strong only through exercise, so do the teeth grow strong only by exercise.

If we can impress our patients with the importance of the thorough mouth-treatment of food as related to general health, and likewise to the health of the teeth, we can perform for them no greater service.

No one has better opportunity for studying the cause and effect of the abnormal conditions of the saliva than we, whose daily work consists so largely in restoring to usefulness the delicate and highly sensitive dental organs, that are constantly bathed in this very interesting fluid.

I realize that this short paper does not do the subject justice; but if the facts that I have tried to bring out should but stimulate any one to further investigation, the object of this paper will have been fulfilled.

